# Nutrient timing revisited: is there a post-exercise anabolic window?

**DOI:** 10.1186/1550-2783-10-5

**Published:** 2013-01-29

**Authors:** Alan Albert Aragon, Brad Jon Schoenfeld

**Affiliations:** 1 California State University, Northridge, CA, USA; 2 Department of Health Science, Lehman College, Bronx, NY, USA

## Abstract

Nutrient timing is a popular nutritional strategy that involves the consumption of combinations of nutrients--primarily protein and carbohydrate--in and around an exercise session. Some have claimed that this approach can produce dramatic improvements in body composition. It has even been postulated that the timing of nutritional consumption may be more important than the absolute daily intake of nutrients. The post-exercise period is widely considered the most critical part of nutrient timing. Theoretically, consuming the proper ratio of nutrients during this time not only initiates the rebuilding of damaged muscle tissue and restoration of energy reserves, but it does so in a supercompensated fashion that enhances both body composition and exercise performance. Several researchers have made reference to an anabolic “window of opportunity” whereby a limited time exists after training to optimize training-related muscular adaptations. However, the importance - and even the existence - of a post-exercise ‘window’ can vary according to a number of factors. Not only is nutrient timing research open to question in terms of applicability, but recent evidence has directly challenged the classical view of the relevance of post-exercise nutritional intake with respect to anabolism. Therefore, the purpose of this paper will be twofold: 1) to review the existing literature on the effects of nutrient timing with respect to post-exercise muscular adaptations, and; 2) to draw relevant conclusions that allow practical, evidence-based nutritional recommendations to be made for maximizing the anabolic response to exercise.

## Introduction

Over the past two decades, nutrient timing has been the subject of numerous research studies and reviews. The basis of nutrient timing involves the consumption of combinations of nutrients--primarily protein and carbohydrate--in and around an exercise session. The strategy is designed to maximize exercise-induced muscular adaptations and facilitate repair of damaged tissue [1]. Some have claimed that such timing strategies can produce dramatic improvements in body composition, particularly with respect to increases in fat-free mass [2]. It has even been postulated that the timing of nutritional consumption may be more important than the absolute daily intake of nutrients [3].

The post-exercise period is often considered the most critical part of nutrient timing. An intense resistance training workout results in the depletion of a significant proportion of stored fuels (including glycogen and amino acids) as well as causing damage to muscle fibers. Theoretically, consuming the proper ratio of nutrients during this time not only initiates the rebuilding of damaged tissue and restoration of energy reserves, but it does so in a supercompensated fashion that enhances both body composition and exercise performance. Several researchers have made reference to an “anabolic window of opportunity” whereby a limited time exists after training to optimize training-related muscular adaptations [3-5].

However, the importance – and even the existence – of a post-exercise ‘window’ can vary according to a number of factors. Not only is nutrient timing research open to question in terms of applicability, but recent evidence has directly challenged the classical view of the relevance of post-exercise nutritional intake on anabolism. Therefore, the purpose of this paper will be twofold: 1) to review the existing literature on the effects of nutrient timing with respect to post-exercise muscular adaptations, and; 2) to draw relevant conclusions that allow evidence-based nutritional recommendations to be made for maximizing the anabolic response to exercise.

### Glycogen repletion

A primary goal of traditional post-workout nutrient timing recommendations is to replenish glycogen stores. Glycogen is considered essential to optimal resistance training performance, with as much as 80% of ATP production during such training derived from glycolysis [6]. MacDougall et al. [7] demonstrated that a single set of elbow flexion at 80% of 1 repetition maximum (RM) performed to muscular failure caused a 12% reduction in mixed-muscle glycogen concentration, while three sets at this intensity resulted in a 24% decrease. Similarly, Robergs et al. [8] reported that 3 sets of 12 RM performed to muscular failure resulted in a 26.1% reduction of glycogen stores in the vastus lateralis while six sets at this intensity led to a 38% decrease, primarily resulting from glycogen depletion in type II fibers compared to type I fibers. It therefore stands to reason that typical high volume bodybuilding-style workouts involving multiple exercises and sets for the same muscle group would deplete the majority of local glycogen stores.

In addition, there is evidence that glycogen serves to mediate intracellular signaling. This appears to be due, at least in part, to its negative regulatory effects on AMP-activated protein kinase (AMPK). Muscle anabolism and catabolism are regulated by a complex cascade of signaling pathways. Several pathways that have been identified as particularly important to muscle anabolism include mammalian target of rapamycin (mTOR), mitogen-activated protein kinase (MAPK), and various calcium- (Ca^2+^) dependent pathways. AMPK, on the other hand, is a cellular energy sensor that serves to enhance energy availability. As such, it blunts energy-consuming processes including the activation of mTORC1 mediated by insulin and mechanical tension, as well as heightening catabolic processes such as glycolysis, beta-oxidation, and protein degradation [9]. mTOR is considered a master network in the regulation of skeletal muscle growth [10,11], and its inhibition has a decidedly negative effect on anabolic processes [12]. Glycogen has been shown to inhibit purified AMPK in cell-free assays [13], and low glycogen levels are associated with an enhanced AMPK activity in humans *in vivo*[14].

Creer et al. [15] demonstrated that changes in the phosphorylation of protein kinase B (Akt) are dependent on pre-exercise muscle glycogen content. After performing 3 sets of 10 repetitions of knee extensions with a load equating to 70% of 1 repetition maximum, early phase post-exercise Akt phosphorylation was increased only in the glycogen-loaded muscle, with no effect seen in the glycogen-depleted contralateral muscle. Glycogen inhibition also has been shown to blunt S6K activation, impair translation, and reduce the amount of mRNA of genes responsible for regulating muscle hypertrophy [16,17]. In contrast to these findings, a recent study by Camera et al. [18] found that high-intensity resistance training with low muscle glycogen levels did not impair anabolic signaling or muscle protein synthesis (MPS) during the early (4 h) postexercise recovery period. The discrepancy between studies is not clear at this time.

Glycogen availability also has been shown to mediate muscle protein breakdown. Lemon and Mullin [19] found that nitrogen losses more than doubled following a bout of exercise in a glycogen-depleted versus glycogen-loaded state. Other researchers have displayed a similar inverse relationship between glycogen levels and proteolysis [20]. Considering the totality of evidence, maintaining a high intramuscular glycogen content at the onset of training appears beneficial to desired resistance training outcomes.

Studies show a supercompensation of glycogen stores when carbohydrate is consumed immediately post-exercise, and delaying consumption by just 2 hours attenuates the rate of muscle glycogen re-synthesis by as much as 50% [21]. Exercise enhances insulin-stimulated glucose uptake following a workout with a strong correlation noted between the amount of uptake and the magnitude of glycogen utilization [22]. This is in part due to an increase in the translocation of GLUT4 during glycogen depletion [23,24] thereby facilitating entry of glucose into the cell. In addition, there is an exercise-induced increase in the activity of glycogen synthase—the principle enzyme involved in promoting glycogen storage [25]. The combination of these factors facilitates the rapid uptake of glucose following an exercise bout, allowing glycogen to be replenished at an accelerated rate.

There is evidence that adding protein to a post-workout carbohydrate meal can enhance glycogen re-synthesis. Berardi et al. [26] demonstrated that consuming a protein-carbohydrate supplement in the 2-hour period following a 60-minute cycling bout resulted in significantly greater glycogen resynthesis compared to ingesting a calorie-equated carbohydrate solution alone. Similarly, Ivy et al. [27] found that consumption of a combination of protein and carbohydrate after a 2+ hour bout of cycling and sprinting increased muscle glycogen content significantly more than either a carbohydrate-only supplement of equal carbohydrate or caloric equivalency. The synergistic effects of protein-carbohydrate have been attributed to a more pronounced insulin response [28], although it should be noted that not all studies support these findings [29]. Jentjens et al. [30] found that given ample carbohydrate dosing (1.2 g/kg/hr), the addition of a protein and amino acid mixture (0.4 g/kg/hr) did not increase glycogen synthesis during a 3-hour post-depletion recovery period.

Despite a sound theoretical basis, the practical significance of expeditiously repleting glycogen stores remains dubious. Without question, expediting glycogen resynthesis is important for a narrow subset of endurance sports where the duration between glycogen-depleting events is limited to less than approximately 8 hours [31]. Similar benefits could potentially be obtained by those who perform two-a-day split resistance training bouts (i.e. morning and evening) provided the same muscles will be worked during the respective sessions. However, for goals that are not specifically focused on the performance of multiple exercise bouts in the same day, the urgency of glycogen resynthesis is greatly diminished. High-intensity resistance training with moderate volume (6-9 sets per muscle group) has only been shown to reduce glycogen stores by 36-39% [8,32]. Certain athletes are prone to performing significantly more volume than this (i.e., competitive bodybuilders), but increased volume typically accompanies decreased frequency. For example, training a muscle group with 16-20 sets in a single session is done roughly once per week, whereas routines with 8-10 sets are done twice per week. In scenarios of higher volume and frequency of resistance training, incomplete resynthesis of pre-training glycogen levels would not be a concern aside from the far-fetched scenario where exhaustive training bouts of the same muscles occur after recovery intervals shorter than 24 hours. However, even in the event of complete glycogen depletion, replenishment to pre-training levels occurs well-within this timeframe, regardless of a significantly delayed post-exercise carbohydrate intake. For example, Parkin et al [33] compared the immediate post-exercise ingestion of 5 high-glycemic carbohydrate meals with a 2-hour wait before beginning the recovery feedings. No significant between-group differences were seen in glycogen levels at 8 hours and 24 hours post-exercise. In further support of this point, Fox et al. [34] saw no significant reduction in glycogen content 24 hours after depletion despite adding 165 g fat collectively to the post-exercise recovery meals and thus removing any potential advantage of high-glycemic conditions.

### Protein breakdown

Another purported benefit of post-workout nutrient timing is an attenuation of muscle protein breakdown. This is primarily achieved by spiking insulin levels, as opposed to increasing amino acid availability [35,36]. Studies show that muscle protein breakdown is only slightly elevated immediately post-exercise and then rapidly rises thereafter [36]. In the fasted state, muscle protein breakdown is significantly heightened at 195 minutes following resistance exercise, resulting in a net negative protein balance [37]. These values are increased as much as 50% at the 3 hour mark, and elevated proteolysis can persist for up to 24 hours of the post-workout period [36].

Although insulin has known anabolic properties [38,39], its primary impact post-exercise is believed to be anti-catabolic [40-43]. The mechanisms by which insulin reduces proteolysis are not well understood at this time. It has been theorized that insulin-mediated phosphorylation of PI3K/Akt inhibits transcriptional activity of the proteolytic Forkhead family of transcription factors, resulting in their sequestration in the sarcoplasm away from their target genes [44]. Down-regulation of other aspects of the ubiquitin-proteasome pathway are also believed to play a role in the process [45]. Given that muscle hypertrophy represents the difference between myofibrillar protein synthesis and proteolysis, a decrease in protein breakdown would conceivably enhance accretion of contractile proteins and thus facilitate greater hypertrophy. Accordingly, it seems logical to conclude that consuming a protein-carbohydrate supplement following exercise would promote the greatest reduction in proteolysis since the combination of the two nutrients has been shown to elevate insulin levels to a greater extent than carbohydrate alone [28].

However, while the theoretical basis behind spiking insulin post-workout is inherently sound, it remains questionable as to whether benefits extend into practice. First and foremost, research has consistently shown that, in the presence of elevated plasma amino acids, the effect of insulin elevation on net muscle protein balance plateaus within a range of 15–30 mU/L [45,46]; roughly 3–4 times normal fasting levels. This insulinogenic effect is easily accomplished with typical mixed meals, considering that it takes approximately 1–2 hours for circulating substrate levels to peak, and 3–6 hours (or more) for a complete return to basal levels depending on the size of a meal. For example, Capaldo et al. [47] examined various metabolic effects during a 5-hour period after ingesting a solid meal comprised of 75 g carbohydrate 37 g protein, and 17 g fat. This meal was able to raise insulin 3 times above fasting levels within 30 minutes of consumption. At the 1-hour mark, insulin was 5 times greater than fasting. At the 5-hour mark, insulin was still double the fasting levels. In another example, Power et al. [48] showed that a 45g dose of whey protein isolate takes approximately 50 minutes to cause blood amino acid levels to peak. Insulin concentrations peaked 40 minutes after ingestion, and remained at elevations seen to maximize net muscle protein balance (15-30 mU/L, or 104-208 pmol/L) for approximately 2 hours. The inclusion of carbohydrate to this protein dose would cause insulin levels to peak higher and stay elevated even longer. Therefore, the recommendation for lifters to spike insulin post-exercise is somewhat trivial. The classical post-exercise objective to quickly reverse catabolic processes to promote recovery and growth may only be applicable in the absence of a properly constructed pre-exercise meal.

Moreover, there is evidence that the effect of protein breakdown on muscle protein accretion may be overstated. Glynn et al. [49] found that the post-exercise anabolic response associated with combined protein and carbohydrate consumption was largely due to an elevation in muscle protein synthesis with only a minor influence from reduced muscle protein breakdown. These results were seen regardless of the extent of circulating insulin levels. Thus, it remains questionable as to what, if any, positive effects are realized with respect to muscle growth from spiking insulin after resistance training.

### Protein synthesis

Perhaps the most touted benefit of post-workout nutrient timing is that it potentiates increases in MPS. Resistance training alone has been shown to promote a twofold increase in protein synthesis following exercise, which is counterbalanced by the accelerated rate of proteolysis [36]. It appears that the stimulatory effects of hyperaminoacidemia on muscle protein synthesis, especially from essential amino acids, are potentiated by previous exercise [35,50]. There is some evidence that carbohydrate has an additive effect on enhancing post-exercise muscle protein synthesis when combined with amino acid ingestion [51], but others have failed to find such a benefit [52,53].

Several studies have investigated whether an “anabolic window” exists in the immediate post-exercise period with respect to protein synthesis. For maximizing MPS, the evidence supports the superiority of post-exercise free amino acids and/or protein (in various permutations with or without carbohydrate) compared to solely carbohydrate or non-caloric placebo [50,51,54-59]. However, despite the common recommendation to consume protein as soon as possible post-exercise [60,61], evidence-based support for this practice is currently lacking. Levenhagen et al. [62] demonstrated a clear benefit to consuming nutrients as soon as possible after exercise as opposed to delaying consumption. Employing a within-subject design,10 volunteers (5 men, 5 women) consumed an oral supplement containing 10 g protein, 8 g carbohydrate and 3 g fat either immediately following or three hours post-exercise. Protein synthesis of the legs and whole body was increased threefold when the supplement was ingested immediately after exercise, as compared to just 12% when consumption was delayed. A limitation of the study was that training involved moderate intensity, long duration aerobic exercise. Thus, the increased fractional synthetic rate was likely due to greater mitochondrial and/or sarcoplasmic protein fractions, as opposed to synthesis of contractile elements [36]. In contrast to the timing effects shown by Levenhagen et al. [62], previous work by Rasmussen et al. [56] showed no significant difference in leg net amino acid balance between 6 g essential amino acids (EAA) co-ingested with 35 g carbohydrate taken 1 hour versus 3 hours post-exercise. Compounding the unreliability of the post-exercise ‘window’ is the finding by Tipton et al. [63] that immediate pre-exercise ingestion of the same EAA-carbohydrate solution resulted in a significantly greater and more sustained MPS response compared to the immediate post-exercise ingestion, although the validity of these findings have been disputed based on flawed methodology [36]. Notably, Fujita et al [64] saw opposite results using a similar design, except the EAA-carbohydrate was ingested 1 hour prior to exercise compared to ingestion immediately pre-exercise in Tipton et al. [63]. Adding yet more incongruity to the evidence, Tipton et al. [65] found no significant difference in net MPS between the ingestion of 20 g whey immediately pre- versus the same solution consumed 1 hour post-exercise. Collectively, the available data lack any consistent indication of an ideal post-exercise timing scheme for maximizing MPS.

It also should be noted that measures of MPS assessed following an acute bout of resistance exercise do not always occur in parallel with chronic upregulation of causative myogenic signals [66] and are not necessarily predictive of long-term hypertrophic responses to regimented resistance training [67]. Moreover, the post-exercise rise in MPS in untrained subjects is not recapitulated in the trained state [68], further confounding practical relevance. Thus, the utility of acute studies is limited to providing clues and generating hypotheses regarding hypertrophic adaptations; any attempt to extrapolate findings from such data to changes in lean body mass is speculative, at best.

### Muscle hypertrophy

A number of studies have directly investigated the long-term hypertrophic effects of post-exercise protein consumption. The results of these trials are curiously conflicting, seemingly because of varied study design and methodology. Moreover, a majority of studies employed both pre- and post-workout supplementation, making it impossible to tease out the impact of consuming nutrients after exercise. These confounding issues highlight the difficulty in attempting to draw relevant conclusions as to the validity of an “anabolic window.” What follows is an overview of the current research on the topic. Only those studies that specifically evaluated immediate (≤ 1 hour) post-workout nutrient provision are discussed (see Table 1 for a summary of data).

**Table 1:** Post-exercise nutrition and muscle hypertrophy

**Study**	**Subjects**	**Supplementation**	**Protein matched with Control?**	**Measurement instrument**	**Training protocol**	**Results**
Esmarck et al. [69]	13 untrained elderly males	10 g milk/soy protein combo consumed either immediately or 2 hours after exercise	Yes	MRI and muscle biopsy	Progressive resistance training consisting of multiple sets of lat pulldown, leg press and knee extension performed 3 days/wk for 12 wk	Significant increase in muscle CSA with immediate vs. delayed supplementation
Cribb and Hayes [70]	23 young recreational male bodybuilders	1 g/kg of a supplement containing 40 g whey isolate, 43 g glucose, and 7 g creatine monohydrate consumed either immediately before and after exercise or in the early morning and late evening	Yes	DXA and muscle biopsy	Progressive resistance training consisting of exercises for the major muscle groups performed 3 days/wk for 10 wks	Significant increases in lean body mass and muscle CSA of type II fibers in immediate vs. delayed supplementation
Willoughby et al. [71]	19 untrained young males	20 g protein or 20 g dextrose consumed 1 hour before and after exercise	No	Hydrostatic weighing, muscle biopsy, surface measurements	Progressive resistance training consisting of 3 sets of 6–8 repetitions for all the major muscles performed 4 days/wk for 10 wks	Significant increase in total body mass, fat-free mass, and thigh mass with protein vs. carb supplementation
Hulmi et al. [72]	31 untrained young males	15 g whey isolate or placebo consumed immediately before and after exercise	No	MRI, muscle biopsy	Progressive, periodized total body resistance training consisting of 2–5 sets of 5–20 repetitions performed 2 days/wk for 21 wks.	Significant increase in CSA of the vastus lateralis but not of the other quadriceps muscles in supplemented group versus placebo.
Verdijk et al. [73]	28 untrained elderly males	10 g casein hydrolysate or placebo consumed immediately before and after exercise	No	DXA, CT, and muscle biopsy	Progressive resistance training consisting of multiple sets of leg press and knee extension performed 3 days/wk for 12 wks	No significant differences in muscle CSA between groups
Hoffman et al. [74]	33 well-trained young males	Supplement containing 42 g protein (milk/collagen blend) and 2 g carbohydrate consumed either immediately before and after exercise or in the early morning and late evening	Yes	DXA	Progressive resistance training consisting of 3–4 sets of 6–10 repetitions of multiple exercises for the entire body peformed 4 days/wk for 10 weeks.	No significant differences in total body mass or lean body mass between groups.
Erskine et al. [75]	33 untrained young males	20 g high quality protein or placebo consumed immediately before and after exercise	No	MRI	4-6 sets of elbow flexion performed 3 days/wk for 12 weeks	No significant differences in muscle CSA between groups

Esmarck et al. [69] provided the first experimental evidence that consuming protein immediately after training enhanced muscular growth compared to delayed protein intake. Thirteen untrained elderly male volunteers were matched in pairs based on body composition and daily protein intake and divided into two groups: P0 or P2. Subjects performed a progressive resistance training program of multiple sets for the upper and lower body. P0 received an oral protein/carbohydrate supplement immediately post-exercise while P2 received the same supplement 2 hours following the exercise bout. Training was carried out 3 days a week for 12 weeks. At the end of the study period, cross-sectional area (CSA) of the quadriceps femoris and mean fiber area were significantly increased in the P0 group while no significant increase was seen in P2. These results support the presence of a post-exercise window and suggest that delaying post-workout nutrient intake may impede muscular gains.

In contrast to these findings, Verdijk et al. [73] failed to detect any increases in skeletal muscle mass from consuming a post-exercise protein supplement in a similar population of elderly men. Twenty-eight untrained subjects were randomly assigned to receive either a protein or placebo supplement consumed immediately before and immediately following the exercise session. Subjects performed multiple sets of leg press and knee extension 3 days per week, with the intensity of exercise progressively increased over the course of the 12 week training period. No significant differences in muscle strength or hypertrophy were noted between groups at the end of the study period indicating that post exercise nutrient timing strategies do not enhance training-related adaptation. It should be noted that, as opposed to the study by Esmark et al. [69] this study only investigated adaptive responses of supplementation on the thigh musculature; it therefore is not clear based on these results whether the upper body might respond differently to post-exercise supplementation than the lower body.

In an elegant single-blinded design, Cribb and Hayes [70] found a significant benefit to post-exercise protein consumption in 23 recreational male bodybuilders. Subjects were randomly divided into either a PRE-POST group that consumed a supplement containing protein, carbohydrate and creatine immediately before and after training or a MOR-EVE group that consumed the same supplement in the morning and evening at least 5 hours outside the workout. Both groups performed regimented resistance training that progressively increased intensity from 70% 1RM to 95% 1RM over the course of 10 weeks. Results showed that the PRE-POST group achieved a significantly greater increase in lean body mass and increased type II fiber area compared to MOR-EVE. Findings support the benefits of nutrient timing on training-induced muscular adaptations. The study was limited by the addition of creatine monohydrate to the supplement, which may have facilitated increased uptake following training. Moreover, the fact that the supplement was taken both pre- and post-workout confounds whether an anabolic window mediated results.

Willoughby et al. [71] also found that nutrient timing resulted in positive muscular adaptations. Nineteen untrained male subjects were randomly assigned to either receive 20 g of protein or 20 grams dextrose administered 1 hour before and after resistance exercise. Training consisted of 3 sets of 6–8 repetitions at 85%–90% intensity. Training was performed 4 times a week over the course of 10 weeks. At the end of the study period, total body mass, fat-free mass, and thigh mass was significantly greater in the protein-supplemented group compared to the group that received dextrose. Given that the group receiving the protein supplement consumed an additional 40 grams of protein on training days, it is difficult to discern whether results were due to the increased protein intake or the timing of the supplement.

In a comprehensive study of well-trained subjects, Hoffman et al. [74] randomly assigned 33 well-trained males to receive a protein supplement either in the morning and evening (n = 13) or immediately before and immediately after resistance exercise (n = 13). Seven participants served as unsupplemented controls. Workouts consisted of 3–4 sets of 6–10 repetitions of multiple exercises for the entire body. Training was carried out on 4 day-a-week split routine with intensity progressively increased over the course of the study period. After 10 weeks, no significant differences were noted between groups with respect to body mass and lean body mass. The study was limited by its use of DXA to assess body composition, which lacks the sensitivity to detect small changes in muscle mass compared to other imaging modalities such as MRI and CT [76].

Hulmi et al. [72] randomized 31 young untrained male subjects into 1 of 3 groups: protein supplement (n = 11), non-caloric placebo (n = 10) or control (n = 10). High-intensity resistance training was carried out over 21 weeks. Supplementation was provided before and after exercise. At the end of the study period, muscle CSA was significantly greater in the protein-supplemented group compared to placebo or control. A strength of the study was its long-term training period, providing support for the beneficial effects of nutrient timing on chronic hypertrophic gains. Again, however, it is unclear whether enhanced results associated with protein supplementation were due to timing or increased protein consumption.

Most recently, Erskine et al. [75] failed to show a hypertrophic benefit from post-workout nutrient timing. Subjects were 33 untrained young males, pair-matched for habitual protein intake and strength response to a 3-week pre-study resistance training program. After a 6-week washout period where no training was performed, subjects were then randomly assigned to receive either a protein supplement or a placebo immediately before and after resistance exercise. Training consisted of 6– 8 sets of elbow flexion carried out 3 days a week for 12 weeks. No significant differences were found in muscle volume or anatomical cross-sectional area between groups.

## Discussion

Despite claims that immediate post-exercise nutritional intake is essential to maximize hypertrophic gains, evidence-based support for such an “anabolic window of opportunity” is far from definitive. The hypothesis is based largely on the pre-supposition that training is carried out in a fasted state. During fasted exercise, a concomitant increase in muscle protein breakdown causes the pre-exercise net negative amino acid balance to persist in the post-exercise period despite training-induced increases in muscle protein synthesis [36]. Thus, in the case of resistance training after an overnight fast, it would make sense to provide immediate nutritional intervention--ideally in the form of a combination of protein and carbohydrate--for the purposes of promoting muscle protein synthesis and reducing proteolysis, thereby switching a net catabolic state into an anabolic one. Over a chronic period, this tactic could conceivably lead cumulatively to an increased rate of gains in muscle mass.

This inevitably begs the question of how pre-exercise nutrition might influence the urgency or effectiveness of post-exercise nutrition, since not everyone engages in fasted training. In practice, it is common for those with the primary goal of increasing muscular size and/or strength to make a concerted effort to consume a pre-exercise meal within 1-2 hours prior to the bout in attempt to maximize training performance. Depending on its size and composition, this meal can conceivably function as both a pre- and an immediate post-exercise meal, since the time course of its digestion/absorption can persist well into the recovery period. Tipton et al. [63] observed that a relatively small dose of EAA (6 g) taken immediately pre-exercise was able to elevate blood and muscle amino acid levels by roughly 130%, and these levels remained elevated for 2 hours after the exercise bout. Although this finding was subsequently challenged by Fujita et al. [64], other research by Tipton et al. [65] showed that the ingestion of 20 g whey taken immediately pre-exercise elevated muscular uptake of amino acids to 4.4 times pre-exercise resting levels during exercise, and did not return to baseline levels until 3 hours post-exercise. These data indicate that even minimal-to-moderate pre-exercise EAA or high-quality protein taken immediately before resistance training is capable of sustaining amino acid delivery into the post-exercise period. Given this scenario, immediate post-exercise protein dosing for the aim of mitigating catabolism seems redundant. The next scheduled protein-rich meal (whether it occurs immediately or 1–2 hours post-exercise) is likely sufficient for maximizing recovery and anabolism.

On the other hand, there are others who might train before lunch or after work, where the previous meal was finished 4–6 hours prior to commencing exercise. This lag in nutrient consumption can be considered significant enough to warrant post-exercise intervention if muscle retention or growth is the primary goal. Layman [77] estimated that the anabolic effect of a meal lasts 5-6 hours based on the rate of postprandial amino acid metabolism. However, infusion-based studies in rats [78,79] and humans [80,81] indicate that the postprandial rise in MPS from ingesting amino acids or a protein-rich meal is more transient, returning to baseline within 3 hours despite sustained elevations in amino acid availability. It thus has been hypothesized that a “muscle full” status can be reached where MPS becomes refractory, and circulating amino acids are shunted toward oxidation or fates other than MPS. In light of these findings, when training is initiated more than ~3–4 hours after the preceding meal, the classical recommendation to consume protein (at least 25 g) as soon as possible seems warranted in order to reverse the catabolic state, which in turn could expedite muscular recovery and growth. However, as illustrated previously, minor pre-exercise nutritional interventions can be undertaken if a significant delay in the post-exercise meal is anticipated.

An interesting area of speculation is the generalizability of these recommendations across training statuses and age groups. Burd et al. [82] reported that an acute bout of resistance training in untrained subjects stimulates both mitochondrial and myofibrillar protein synthesis, whereas in trained subjects, protein synthesis becomes more preferential toward the myofibrillar component. This suggests a less global response in advanced trainees that potentially warrants closer attention to protein timing and type (e.g., high-leucine sources such as dairy proteins) in order to optimize rates of muscular adaptation. In addition to training status, age can influence training adaptations. Elderly subjects exhibit what has been termed “anabolic resistance,” characterized by a lower receptivity to amino acids and resistance training [83]. The mechanisms underlying this phenomenon are not clear, but there is evidence that in younger adults, the acute anabolic response to protein feeding appears to plateau at a lower dose than in elderly subjects. Illustrating this point, Moore et al. [84] found that 20 g whole egg protein maximally stimulated post-exercise MPS, while 40 g increased leucine oxidation without any further increase in MPS in young men. In contrast, Yang et al. [85] found that elderly subjects displayed greater increases in MPS when consuming a post-exercise dose of 40 g whey protein compared to 20 g. These findings suggest that older subjects require higher individual protein doses for the purpose of optimizing the anabolic response to training. Further research is needed to better assess post-workout nutrient timing response across various populations, particularly with respect to trained/untrained and young/elderly subjects.

The body of research in this area has several limitations. First, while there is an abundance of acute data, controlled, long-term trials that systematically compare the effects of various post-exercise timing schemes are lacking. The majority of chronic studies have examined pre- and post-exercise supplementation simultaneously, as opposed to comparing the two treatments against each other. This prevents the possibility of isolating the effects of either treatment. That is, we cannot know whether pre- or post-exercise supplementation was the critical contributor to the outcomes (or lack thereof). Another important limitation is that the majority of chronic studies neglect to match total protein intake between the conditions compared. As such, it’s not possible to ascertain whether positive outcomes were influenced by timing relative to the training bout, or simply by a greater protein intake overall. Further, dosing strategies employed in the preponderance of chronic nutrient timing studies have been overly conservative, providing only 10–20 g protein near the exercise bout. More research is needed using protein doses known to maximize acute anabolic response, which has been shown to be approximately 20–40 g, depending on age [84,85]. There is also a lack of chronic studies examining the co-ingestion of protein and carbohydrate near training. Thus far, chronic studies have yielded equivocal results. On the whole, they have not corroborated the consistency of positive outcomes seen in acute studies examining post-exercise nutrition.

Another limitation is that the majority of studies on the topic have been carried out in untrained individuals. Muscular adaptations in those without resistance training experience tend to be robust, and do not necessarily reflect gains experienced in trained subjects. It therefore remains to be determined whether training status influences the hypertrophic response to post-exercise nutritional supplementation.

A final limitation of the available research is that current methods used to assess muscle hypertrophy are widely disparate, and the accuracy of the measures obtained are inexact [68]. As such, it is questionable whether these tools are sensitive enough to detect small differences in muscular hypertrophy. Although minor variances in muscle mass would be of little relevance to the general population, they could be very meaningful for elite athletes and bodybuilders. Thus, despite conflicting evidence, the potential benefits of post-exercise supplementation cannot be readily dismissed for those seeking to optimize a hypertrophic response. By the same token, widely varying feeding patterns among individuals challenge the common assumption that the post-exercise “anabolic window of opportunity” is universally narrow and urgent.

### Practical applications

Distilling the data into firm, specific recommendations is difficult due to the inconsistency of findings and scarcity of systematic investigations seeking to optimize pre- and/or post-exercise protein dosage and timing. Practical nutrient timing applications for the goal of muscle hypertrophy inevitably must be tempered with field observations and experience in order to bridge gaps in the scientific literature. With that said, high-quality protein dosed at 0.4–0.5 g/kg of LBM at both pre- and post-exercise is a simple, relatively fail-safe general guideline that reflects the current evidence showing a maximal acute anabolic effect of 20–40 g [53,84,85]. For example, someone with 70 kg of LBM would consume roughly 28–35 g protein in both the pre- and post exercise meal. Exceeding this would be have minimal detriment if any, whereas significantly under-shooting or neglecting it altogether would not maximize the anabolic response.

Due to the transient anabolic impact of a protein-rich meal and its potential synergy with the trained state, pre- and post-exercise meals should not be separated by more than approximately 3–4 hours, given a typical resistance training bout lasting 45–90 minutes. If protein is delivered within particularly large mixed-meals (which are inherently more anticatabolic), a case can be made for lengthening the interval to 5–6 hours. This strategy covers the hypothetical timing benefits while allowing significant flexibility in the length of the feeding windows before and after training. Specific timing within this general framework would vary depending on individual preference and tolerance, as well as exercise duration. One of many possible examples involving a 60-minute resistance training bout could have up to 90-minute feeding windows on both sides of the bout, given central placement between the meals. In contrast, bouts exceeding typical duration would default to shorter feeding windows if the 3–4 hour pre- to post-exercise meal interval is maintained. Shifting the training session closer to the pre- or post-exercise meal should be dictated by personal preference, tolerance, and lifestyle/scheduling constraints.

Even more so than with protein, carbohydrate dosage and timing relative to resistance training is a gray area lacking cohesive data to form concrete recommendations. It is tempting to recommend pre- and post-exercise carbohydrate doses that at least match or exceed the amounts of protein consumed in these meals. However, carbohydrate availability during and after exercise is of greater concern for endurance as opposed to strength or hypertrophy goals. Furthermore, the importance of co-ingesting post-exercise protein and carbohydrate has recently been challenged by studies examining the early recovery period, particularly when sufficient protein is provided. Koopman et al [52] found that after full-body resistance training, adding carbohydrate (0.15, or 0.6 g/kg/hr) to amply dosed casein hydrolysate (0.3 g/kg/hr) did not increase whole body protein balance during a 6-hour post-exercise recovery period compared to the protein-only treatment. Subsequently, Staples et al [53] reported that after lower-body resistance exercise (leg extensions), the increase in post-exercise muscle protein balance from ingesting 25 g whey isolate was not improved by an additional 50 g maltodextrin during a 3-hour recovery period. For the goal of maximizing rates of muscle gain, these findings support the broader objective of meeting total daily carbohydrate need instead of specifically timing its constituent doses. Collectively, these data indicate an increased potential for dietary flexibility while maintaining the pursuit of optimal timing.

## Competing interests

The authors declare that they have no competing interests.

## Authors’ contribution

AAA and BJS each contributed equally to the formulation and writing of the manuscript. Both authors read and approved the final manuscript.
